# Inasmuch

**Published:** 1919-11-01

**Authors:** 


### "Inasmuch."

WHEN our days and nights were shadowed by the cruel hand of War,
As it, unrelenting, reaped its harvest red,
And we knew when cannon rumbled and re-echoed on our shore
They were bringing in the wounded and the dead.

Then we set our teeth, determined every one to do his bit,
For we could not fail our heroes over there,
And the Mothers hid their heartache as they bent to sew or knit,
Every stitch to count a deep unuttered prayer.

While the Red Cross banner floated over hospital and hall,
There were shattered limbs to bandage, wounds to dress;
Tho' we gave our best to help them, they were sacrificing all,
And we could not offer one iota less.

Now the dreary days are over, and our maimed and crippled men
Are learning lives of usefulness to live;
But as we turn to comfort and to pleasure once again
Let us not forget the blessed art—to give.

For the needy, the downtrodden, and the sick are always here,
And they crave the kindly word, the healing touch.
Can we pass them by unnoticed, while the message strong and clear
Is resounding through the ages—"Inasmuch"?

Are they worthy? Oh! What matter! Let their merit be their need.
'Tis our part to cheer their lot, not question it,
And as long as there are sick to nurse, and hungry poor to feed,
For the least of them, let's do our little bit.

We have laid aside our knitting, and our flags are packed away,
For their day is done, and we so soon forget.
But the long white wards are crowded still, and need our help to-day
To protect them from the heavy load of debt.

SISTER BEE.

